# Biohybrid Photonic Platform for Subcellular Stimulation and Readout of In Vitro Neurons

**DOI:** 10.1002/advs.202304561

**Published:** 2024-01-02

**Authors:** Corinna Kaspar, Alexander Ivanenko, Julia Lehrich, Jürgen Klingauf, Wolfram H.P. Pernice

**Affiliations:** ^1^ Institute of Physics University of Muenster Heisenbergstr. 11 48149 Muenster Germany; ^2^ Center for Soft Nanoscience University of Muenster Busso‐Peuss‐Str. 10 48149 Muenster Germany; ^3^ Institute of Medical Physics and Biophysics University of Muenster Robert‐Koch‐Str. 31 48149 Muenster Germany; ^4^ Kirchhoff‐Institut for Physics Heidelberg University Im Neuenheimer Feld 227 69120 Heidelberg Germany

**Keywords:** biohybrid devices, cultured neurons, Nanophotonic circuits, optical stimulation

## Abstract

Targeted manipulation of neural activity via light has become an indispensable tool for gaining insights into the intricate processes governing single neurons and complex neural networks. To shed light onto the underlying interaction mechanisms, it is crucial to achieve precise control of individual neural activity, as well as a spatial read‐out resolution on the nanoscale. Here, a versatile photonic platform with subcellular resolution for stimulation and monitoring of in‐vitro neurons is demonstrated. Low‐loss photonic waveguides are fabricated on glass substrates using nanoimprint lithography and featuring a loss of only ‐0.9 ± 0.2 dB cm^−1^ at 489 nm and are combined with optical fiber‐based waveguide‐access and backside total internal reflection fluorescence microscopy. Neurons are grown on the bio‐functionalized photonic chip surface and, expressing the light‐sensitive ion channel Channelrhodopsin‐2, are stimulated within the evanescent field penetration depth of 57 nm of the biocompatible waveguides. The versatility and cost‐efficiency of the platform, along with the possible subcellular resolution, enable tailor‐made investigations of neural interaction dynamics with defined spatial control and high throughput.

## Graphical abstract

Light‐based manipulation of neural activity is vital for understanding single neurons and complex networks. Precise control and nanoscale spatial resolution are essential. A photonic platform using low‐loss waveguides on glass substrates and optical fiber‐based technology enables subcellular stimulation and monitoring of in vitro neurons. This versatile, cost‐effective platform allows tailored studies of neural interaction dynamics with precise spatial control and high throughput.

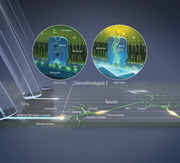

## 1 Introduction

Unraveling the highly complex processes of cognition, behavior, or emotions, efficiently curing the increasing number of neural disorders, or building high‐performing artificial mimics of the human brain requires a profound comprehension of the nervous system's function. Obtaining detailed insight into the encoding mechanisms of a single neuron but also understanding their sophisticated interplay in a multidimensional neural network is a key goal of research in neuroscience. Optogenetics has revolutionized neuroscience by providing a powerful tool to modulate the activity of excitable cells via light exposure.^[^ ^1^, ^2^ ^]^ The underlying principle is the genetic introduction of a light‐sensitive protein into the cell membrane acting as an ion channel. Thus, cross‐membrane currents can be evoked by triggering the opening of the channel with light pulses. If a sufficient number of channels is activated, depolarization or hyperpolarization (depending on the channels’ characteristics) results, and cell activity can be controlled.^[^ ^3^, ^4^, ^5^, ^6^ ^]^ A major advantage of optogenetics compared to the electrical stimulation of neurons is the possibility of cell‐type specific channel expression via genetic engineering. In this way, the stimulation can be restricted to a targeted group of neurons, while surrounding non‐expressing neurons are hardly affected even if they are subject to light irradiation.^[^ ^7^ ^]^ In practice, a straightforward approach is the wide‐field illumination of multiple cells with visible light through an objective^[^ ^8^ ^]^ or an optical fiber.^[^ ^9^, ^10^, ^11^ ^]^ In addition, so‐called optoelectronic probes, i.e., needle‐shaped silicon‐based chips with photonic circuitries or light‐emitting diodes, are frequently used for in‐vivo experiments.^[^ ^12^, ^13^, ^14^, ^15^ ^]^ These have the advantage that several out‐coupling structures enable cell stimulation at distinct positions in the tissue. A precise investigation of neural computation, however, necessitates precise single‐cell stimulation and readout, if not even subcellular resolution. Thus, advanced approaches have been developed to guide light directly to a desired cell without the stimulation of off‐target cells and 2‐photon patterning has been employed to achieve dynamic placement of bioactive cues for spatial cell guidance within a three‐dimensional (3D) hydrogel.^[^ ^16^ ^]^ Spatial light modulators or acoustic‐optic deflectors are used to project computer‐generated holograms into 3D tissue.^[^ ^17^, ^18^, ^19^, ^20^ ^]^ The precisely shaped light pattern enables local stimulation of selected cells with nearly diffraction‐limited spots.^[^ ^21^, ^22^ ^]^ A drawback is the needed expensive and complex equipment as well as the limited speed at which 3D light patterns can be projected with the corresponding setup.^[^ ^21^ ^]^ With the use of a spatial light modulator (SLM), typically achieved temporal resolution is in the order of milliseconds. When digital mirror devices are used, modulation rates > 10 kHz are feasible. Different in‐vitro approaches to achieve single‐cell stimulation have also been demonstrated, including specially designed chips where neurons were stimulated via silicon nitride grating couplers ^[^ ^23^ ^]^ or organic light‐emitting diodes, ^[^ ^24^ ^]^ while their activity was recorded with an array of electrodes on the same chip or the patch clamp technique, respectively. While both experiments showed single‐cell stimulation, the read‐out method has either low resolution or is cumbersome and not scalable.

To reveal coherence between neural code and behaviour, large‐scale investigation of neural activity and, at the same time, the possibility of modulating single neurons with high spatial and temporal accuracy is needed. Here, we introduce a cost‐effective photonic platform for high‐resolution optogenetic stimulation and monitoring of neural activities. Channelrhodopsin‐2 transduced neural cells grow in close vicinity to the surface of photonic waveguides and are locally stimulated by the evanescent field within the overlapping area. The design of the photonic circuits can be flexibly adjusted with waveguide widths much smaller than the cell dimensions. Since the stimulation light is strongly confined to the waveguides, it can be guided to individual neurons or neurites on the chip allowing for targeted neuromodulation with resolution dictated by the waveguide dimensions. Employing a transparent substrate allows us to monitor large‐scale neural activity and enables at the same time subcellular resolution read‐out.

## 2 Waveguide–Neuron Interface for Optogenetic Stimulation

**Figure 1:**
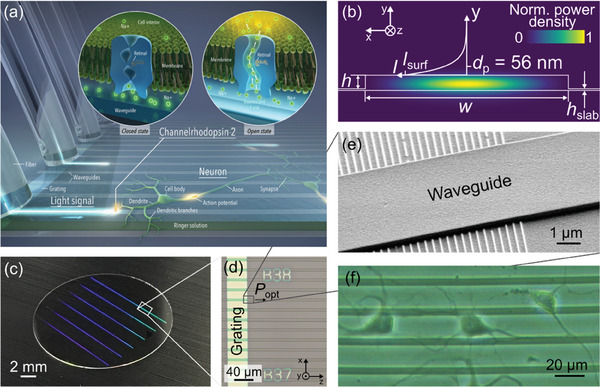
Photonic platform for optogenetic stimulation of neurons. a) Illustration of the waveguide‐neuron interface. Channelrhodopsin‐2 expressing neural cells (depicted in green) grow in close vicinity to the surface of photonic waveguides (depicted in bright grey) and are stimulated by the evanescent field (shown in the magnification). b) Schematic cross‐section of a waveguide guiding the fundamental TE‐like mode (color‐coded normalized power density). The core material is the hybrid polymer OrmoClear, the substrate material is fused silica, and water serves as cladding. The rib waveguide has a width *w* = 4.5 µm, a height *h* = 300 nm, and a slab height of 40 nm. The small aspect ratio was chosen to allow neurons to cross the waveguide architectures and grow in an unrestricted manner. The exponential decay of the evanescent field with its penetration depth *d*_p_ is plotted in *y*‐direction. Note that the penetration depth in the plot was increased for better visibility. The penetration depth is 56 nm for the first three TE‐like modes and varies only slightly due to the low height of the waveguide. For an input power of 1 mW, the computed power density at the surface of the waveguide *I*_surf_ peaks at ≈3.3e5 mW mm^−2^. This very high value is attributed to the strong power confinement in the waveguide with a microscale cross‐section and is sufficient to trigger light‐sensitive transmembrane channels such as channelrhodopsin‐2. c) Photograph of a microscope cover glass with Al gratings diffracting blue light. d) Microscope image showing the principle photonic design. Perpendicularly to the gratings, equidistant straight waveguides are printed on top of the sample, which guides the coupled light (*P*_opt_) to neurons of interest. e) Magnified image of the waveguide on the grating. (f) Neurons growing on waveguides.

The principle of the photonic platform is illustrated in **Figure** **1**a. The key component is a photonic chip with low‐loss waveguides designed for wavelengths ≈488 nm. Genetically modified neurons expressing the light‐sensitive ion channel channelrhodopsin‐2 (ChR‐2) are grown in an aqueous environment directly on top of the photonic structures. The evanescent field of the waveguides interacts with the transduced cells. Hence, at sufficient power levels, ChR‐2 is triggered by the guided light, which causes currents across the cell membrane and prompts the neurons to fire. To enable simultaneous optical monitoring of the neural activity with high resolution from underneath, the photonic structures are realized on a transparent fused silica chip or standard glass coverslip for microscopy.

A schematic cross‐section of the photonic waveguides used for neuron stimulation in our experiments is shown in Figure 1b. The core material is the hybrid polymer OrmoClear (refractive index *n_*OrmoClear = 1.566 @ *λ* = 491 nm) which allows for low‐loss light propagation in the visible wavelength range. The width *w* of the waveguide is set to 4.5 µm and the total height is 340 nm, including a 40 nm high slab induced by the fabrication process (see Methods section). The low aspect ratio was chosen in order to allow the cells to cross waveguides and grow in an unrestricted manner^[^ ^25^ ^]^ and at the same time ensure highly efficient fibre‐to‐chip coupling (for details see Methods section). We chose to minimize the waveguide height in order to allow the cells to cross the devices. At the same time, the waveguide height has to be sufficiently high to provide good confinement of the guided mode. Based on experiments with different waveguide geometries and on a numerical mode analysis we found that a height of 300 nm is a good compromise between these two opposing goals. The color code in Figure 1b represents the simulated normalized power density of the fundamental transverse electric (TE)‐like mode for a wavelength of 491 nm. While the intensity at the sidewalls is comparatively low, the mode strongly overlaps with the waveguide's top surface. This strong overlap is favored by the very low height of the waveguide and the low index‐contrast Δ = (*n*_OrmoClear‐*n*_water)/*n*_OrmoClear between water cladding (*n_*water = 1.340 @ *λ* = 491 nm) and OrmoClear core of 0.144. Outside of the waveguide, the evanescent power density decays exponentially with a penetration depth *d*_p_, i.e., the distance at which the intensity has declined to 1/e of its maximum value.^[^ ^26^ ^]^ The exponentially decaying power density in *y*‐direction away from the waveguide surface is plotted qualitatively above the waveguide (note that in the plot the penetration depth is increased for visualization purposes). The absolute value of *d*_p_ for the first three TE‐like modes amounts ≈56 nm, i.e., due to the low aspect ratio of the waveguide, the three modes penetrate to a similar extent into the water cladding. Since the waveguide has dimensions on the nm to µm scale, the optical mode is tightly confined and very high power densities are achieved with moderate input power. The maximum power densities at the level of the waveguide surface are calculated to be ≈3.3e5 mW mm^−2^ for TE00, TE10, and TE20 with a fixed input power of 1 mW (see Figure S1, Supporting Information). According to Zhang et al,^[^ ^27^ ^]^ a power density of more than 5 mW mm^−2^ is required to successfully trigger ChR‐2. Thus, with an input power in the waveguide of only 1 mW, this threshold is exceeded by five orders of magnitude at the waveguide surface. Given the exponential decay of the evanescent field, the intensity level falls below the threshold of 5 mW mm^−2^ at a distance of 610 nm. Consequently, ChR‐2 located up to this height can still be triggered. The *y*‐location of this intensity threshold can be simply adjusted by changing I_surf_, which varies linearly with the input power in the waveguide. The minimum needed input power to achieve 5 mW mm^−2^ exactly on the surface of the waveguide is ≈15 µW. In our previous work, we studied the approach described in the current manuscript using numerical simulations. The simulations showed that with a power in the range of microwatts in the waveguide, a ChR‐2‐expressing neuron can be stimulated by the evanescent field.^[^ ^28^ ^]^ This extremely low power is beneficial in multiple ways. First, it reduces the risk of phototoxicity to a significant extent, and potential heat input to the sample is decreased and restricted to the waveguide area. This is important since a heat input can affect the physiological processes of the cells or even damage them leading to convoluted measurement results. In addition, the intensity of scattered light is lower which avoids the risk of undesired stimulation of cells in the vicinity, and bleaching of fluorescent markers is prevented. Further, there is no need for expensive powerful light sources.

## 3 Low‐Loss Photonic Platform for 488 nm Wavelength

The simulated photonic waveguides are embedded experimentally in a low‐cost platform. A basic requirement of the photonic platform is the compatibility with biological cells and the overall measurement procedure. This means the photonic devices must be sustained in an aqueous environment, which is essential for living cells. When growing the neurons in close vicinity to the surface of the waveguides, the water‐based culture medium also serves as a cladding. The neurons used in this work are genetically modified to express ChR‐2. This transmembrane channel is sensitive to light at a wavelength ≈488 nm and opens upon exposure. In order to specifically trigger channels with the help of the evanescent field of the waveguide, light of this wavelength must be guided across extended distances on the chip in a confined way and with low loss. The response of the neurons can then be captured via the objective of an inverted microscope. This enables the cells to be measured in a large volume with total‐internal reflection fluorescence (TIRF) microscopy but at the same time requires a transparent substrate.

The activity of the neurons is monitored with the red‐fluorescent dye Rhod‐3, which detects the change in calcium concentration caused by electrical activity.^[^ ^29^ ^]^ The excitation of the marker with a wavelength of 561 nm is realized with an objective from underneath the transparent photonic platform. The response signal is captured with the same objective and directed onto a camera sensor. This configuration has the advantage that high resolution readout is possible. Depending on the culture density and stimulation regime, calcium indicators allow imaging of individual cells and subcellular structures.^[^ ^30^ ^]^ At the same time, the dynamics of multiple neurons present in the camera's field of view can be recorded.

A popular material platform for photonic integrated circuits (PICs) in the visible wavelength range is based on silicon nitride (SiN).^[^ ^31^, ^32^, ^33^, ^34^, ^35^ ^]^ Waveguide propagation losses of <–2.8 dB cm^−1^ in a wavelength range of 446–550 nm have been reported ^[^ ^32^ ^]^ on a silicon substrate. Also tantalum pentoxide (Ta_2_O_5_) is increasingly used^[^ ^36^, ^37^ ^]^ with related propagation losses of <–5 dB cm^−1^ at a wavelength < 500 nm.^[^ ^38^ ^]^ The challenge in the present work is the comparatively short target wavelength of 488 nm in combination with the desired transparent substrate. Deposition of the waveguiding layer on fused silica requires different process parameters compared to silicon, which often leads to a decreased optical quality.^[^ ^35^ ^]^ To overcome this issue, we chose the hybrid polymer Ormocer, which is commercially available in various compositions.^[^ ^39^ ^]^ Ormocer offers low absorption in the visible wavelength range and forms in combination with water a low index contrast system, which further reduces scattering losses.^[^ ^40^ ^]^ In addition, the possibility of patterning via nanoimprint lithography (NIL) offers easy and cost‐effective fabrication and a mostly independent choice of the used substrate and the biocompatibility of the material has been shown for various cell lines.^[^ ^41^, ^42^, ^43^, ^44^ ^]^ Throughout this work, both the hybrid polymer OrmoComp and OrmoClear are used. If descriptions apply to both compositions, the term Ormocer is used. The photonic platform offers additional flexibility regarding the design of the photonic circuit. The entire photonics toolbox is on hand. Since the stimulation light to trigger ChR‐2 is confined to the waveguide, very precise spatial stimulation of individual neurons of the same type or even parts of neurons can be achieved. We realize the waveguides using the fabrication procedure described in the Methods section.

**Figure 2:**
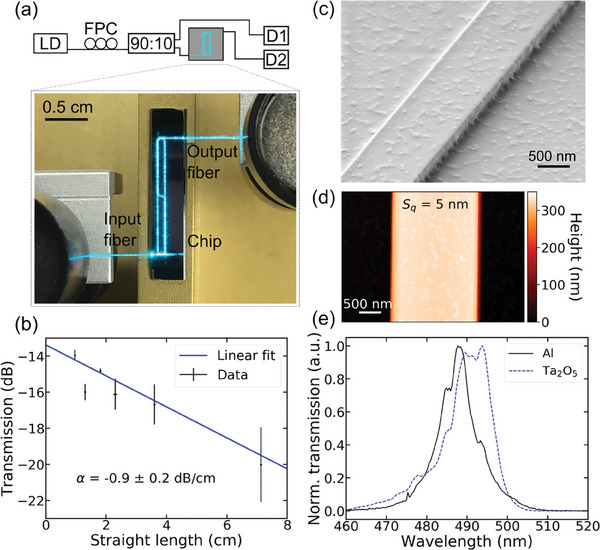
Characterization of low‐loss OrmoComp waveguides at 489 nm. a) Scheme of the setup used to measure propagation losses consisting of a fiber‐coupled laser diode (LD) emitting at 489 nm, a fiber polarization controller (FPC), a 90:10 fiber splitter, and two detectors for reference and signal (D1, D2), respectively. The photograph shows the transmission of light through an 8 cm‐long spiral. b) Transmission plotted versus the length of straight waveguide segments of different spirals. The linear fit yields a propagation loss of −0.9 ± 0.2 dB/cm at a wavelength of 489 nm. c) Scanning electron micrograph of an OrmoComp waveguide fabricated by NIL d) Atomic force micrograph of the surface of a 300 nm high waveguide, exhibiting an RMS area roughness of 5 nm. e) Normalized transmission spectra of a waveguide stamped on a 200 nm high Ta_2_O_5_ grating with a period *p* = 310 nm and a fill factor *ff* = 0.3 and a 40 nm high Al grating with a period *p* = 350 nm and a fill factor *ff* = 0.5, respectively.

In order to couple light into the on‐chip waveguides from an optical fiber, suitable coupling methods have been implemented. In combination with the microscope setup described later on in this work, edge‐coupling is not feasible, since the edges of the sample are not accessible. For this reason, also out‐of‐plane coupling via gratings is used. In order to increase the coupling strength, either Ta_2_O_5_ or aluminum (Al) is used for high‐index gratings underneath the Ormocer waveguides as shown in the inset of Figure 1c. The patterning of the gratings is done via electron beam lithography with a negative tone resist. The exposure of nanoscale features on non‐conductive substrates is challenging due to strong charging effects. To enable a proper electrical discharge, the 40 nm thick Al layer of the later grating is connected to the sample holder. In the case of the also nonconducting Ta_2_O_5_ grating, an additional 10 nm thick sputtered Al layer underneath the resist serves as a conductive layer, which is etched away during the development step with the alkaline developer. The Ormocer waveguide is stamped on top of the gratings, which extend over the entire length of the chip since no alignment on the microscale is possible with the used NIL tool (see Figure 1c). For further fabrication details refer to the Supporting Information (SI). **Figure** **2**e shows the normalized transmission spectra of a Ta_2_O_5_ (period 310 nm, fill factor 0.3) and an Al (period 350 nm, fill factor 0.5) grating, respectively. Coupling losses of –17 dB/grating for the Al grating and –19 dB/grating for the Ta_2_O_5_ grating are achieved. A key advantage of the Al grating is that standard glass coverslips for microscopy can be used as substrate instead of expensive fused silica wafers with deposited functional layers such as Ta_2_O_5_. This fact makes cutting and thinning processes of the sample redundant and additionally reduces the costs substantially.

Propagation losses of the Ormocer waveguides are determined by the cut‐back method. For this, the transmission of spiral‐shaped waveguides with total lengths varying from 1.2 to 8.1 cm is measured. The corresponding setup is illustrated in Figure 2a. The output of a fiber‐coupled laser diode emitting at a wavelength of 489 nm is connected to a fiber polarization controller and a 90:10 fiber splitter. 10% of the signal is guided to a reference detector, while the other part is coupled into the waveguide via edge‐coupling. The signal at the output facet of the waveguide is coupled into a cleaved fiber and measured with a detector. All used fibres are single‐mode. In the photograph in Figure 2a, it is apparent that the light is guided through the entire 8 cm‐long spiral and the elongated shape of the device can be identified. This design allows to separation of the propagation loss in the straight waveguide segments from the loss in the bends. To ensure single‐mode propagation, the waveguide width in the bending regions is 1.2 µm, while the straight segments are 1.8 µm wide. Figure 2b shows a plot of the total measured loss as a function of the straight length of the devices. From the linear fit, a propagation loss of –0.9 ± 0.2 dB cm^−1^ can be extracted. The *y*‐intercept amounts –13.4 ± 0.4 dB and comprises both the coupling loss of the two facets and the bending loss. With the measured coupling loss of – 5.5 dB/facet, the bending loss can be estimated to be –2.4 dB or –0.6 dB/360° with a radius of 300 µm, which is in the same order of magnitude as Lorang et al.^[^ ^45^ ^]^ reported for OrmoClear waveguides.

The material's intrinsic absorption is supposed to be small.^[^ ^39^, ^46^ ^]^ Therefore, we assume that the most dominant source of loss is scattering from rough surfaces. Figure 2d shows an atomic force microscopy (AFM) scan of the surface of an Ormocer waveguide with a root mean square (RMS) surface roughness of roughly 5 nm. The roughness of both the waveguide top surface and sidewalls can also be clearly seen in the scanning electron micrograph in Figure 2c. An order of magnitude estimation can be derived using the analytical model by Payne and Lacey, ^[^ ^40^ ^]^ which relates the loss induced by scattering to the surface roughness of the waveguide (compare SI). The very low propagation loss at these short wavelengths thus enables the realization of large photonic circuits, which are needed to flexibly interrogate cells growing on the chips. In addition, the low scattering loss in combination with the extremely low required input power to stimulate the neurons as suggested by the simulations assures that only cells in direct contact with the waveguide will be affected by the guided optical power.

## 4 Evanescent Field Interaction with Fluorescent Beads

**Figure 3:**
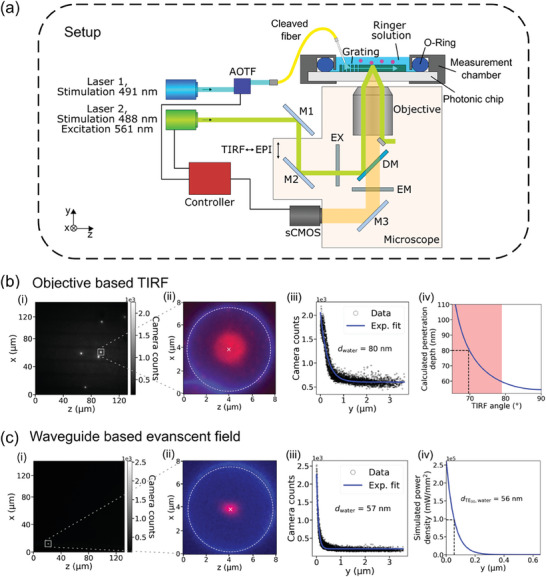
Determination of the evanescent field penetration depth using fluorescent beads. a) Simplified optical setup with inverted microscope body. Laser 1 is coupled into a fiber with the help of an acousto‐optic tuneable filter (AOTF). The cleaved fiber is aligned relative to a coupling grating on the photonic chip, which is mounted into a sealed measurement chamber, to avoid leaking of the Ringer solution. Laser 2 is directed through an excitation filter (EX) to the microscope objective. By changing the position of mirror M2, it can be switched between total internal reflection fluorescence (TIRF) microscopy (shown in the schematic) and epifluorescence microscopy mode. Emitted light from the sample (depicted in orange) travels through the dielectric mirror (DM) and emission filter (EM) and is directed onto the camera sensor. A controller synchronizes the modulation of the two lasers and the camera according to the measurement protocol. b) FITC‐covered microbeads excited with 488 nm via the objective (Laser 2) in TIRF mode. Several beads can be seen in the full camera field of view i). A magnification of a single bead is shown in panel ii), where the TIRF excitation (red channel) is overlapped with the epifluorescence excitation (blue channel), showing the equator of the bead. Camera counts of the red channel in (ii) are plotted over the height y in panel iii). The penetration depth of the evanescent field can be extracted from an exponential fit and amounts 80 nm. Panel iv) illustrates the dependence of the penetration depth (decayed by 1/e) on the incident angle specifically for the used material system and setup. The theoretically achievable range is indicated by the red‐shaded area. c) FITC‐covered microbeads excited with 491 nm (Laser1) via the evanescent field of the waveguide. The penetration depth of the evanescent field of the waveguide amounts 57 nm (panel (iii)). Panel (iv) shows the simulated evanescent field of a waveguide with the same dimensions. The penetration depth amounts 56 nm.

In order to assess the properties of the photonic platform for cell stimulation and, in particular, the interaction range and strength of the evanescent field, the fabricated waveguides were investigated in an aqueous environment. Micrometer‐sized silica beads covered with fluorescein isothiocyanate (FITC) in an index‐matched sucrose solution were dispensed on the photonic waveguides. FITC emits a green fluorescence signal upon excitation at 491 nm. Thus, when the beads are in close vicinity to the waveguide, FITC on the surface is excited by the evanescent field. Using the known diameter of the spherical beads, we quantify the fluorescence in dependency on the vertical distance from the waveguide surface. In this way, the penetration depth of the evanescent field can be determined.^[^ ^47^, ^48^ ^]^ We use the setup shown in **Figure** **3**a, which comprises the body of an inverted microscope as a central part. A laser with a wavelength of 491 nm (Laser 1, illustrated in blue) is coupled into a fiber with the help of an acousto‐optic tuneable filter (AOTF). The cleaved end‐facet of the fiber is placed above the coupling grating of the photonic chip so that light is coupled into the waveguide. The chip is mounted in a sealed measurement chamber, to avoid leakage of the dispensed solution. From the sample emitted light (depicted in orange) is captured by the microscope objective located underneath the transparent chip and is directed onto the camera sensor. Any excitation light is filtered out by the emission filter (EM). The laser and camera can be operated with a controller.

To calibrate the measurement results by fiber excitation, we first carry out a reference measurement, in which the FITC is excited by the evanescent field generated at the photonic chip surface via the TIRF mode of the microscope. For this, the path of a laser emitting at 488 nm (Laser 2, illustrated in green) is adjusted so that it leaves the microscope objective under an angle. If this angle is large enough, total internal reflection occurs at the interface of the photonic chip and the aqueous solution, which leads to an evanescent field penetrating into the liquid. Figure 3b shows the results of the FITC‐excitation through the microscope objective in TIRF mode. The field of view (FOV) of the camera has a size of 132 x 132 µm, while the diameter of the on‐chip surface‐focused laser beam with a 100×‐magnification objective is ≈250 µm. As a consequence, all six beads situated in the FOV are excited and visible in Panel (i). Panel (ii) shows a magnified extract of a single bead. The used beads have a diameter of 7.38 µm. Due to the faster exponential decay of the evanescent field, only fluorophores close to the sample surface, i.e., sitting on the lower hemisphere of the beads, are excited (plotted in the red channel in Panel (ii)). The evanescent field excitation is overlapped with the fluorescence response in epi‐illumination (blue channel). In the latter measurement, the focus is adjusted to the equatorial plane of the bead so that it appears as a ring.

In order to determine the *y* profile of the evanescent field, the fluorescence response has to be related to the distance of the corresponding fluorophore to the sample surface. Based on the surface geometry of the spherical beads, the respective height for each pixel in the two‐dimensional (2D) image is calculated. For this, a Gaussian function is fitted to the intensity distribution in Panel (ii) in order to find the center of the bead, which is represented by the white cross. The white dashed line shows the corresponding diameter of the bead which is in good agreement with the measured equator (blue ring). The position of each pixel relative to the center is converted into its height *y*.^[^ ^48^ ^]^ The measured counts of each pixel are plotted as a function of *y* in Panel (iii). For simplification, we assume that the objective collects the same fraction of fluorescence response for all fluorophores independent of the distance y. In this case, the registered camera counts are proportional to the emitted fluorescence signal of the FITC and, thus, also to the evanescent field intensity.^[^ ^48^ ^]^ The penetration depth can then be extracted for the sucrose solution via an exponential fit of the data plotted in Panel (iii) and then calculated for a water cladding,^[^ ^47^ ^]^ which results in *d*_p_ = 80 nm. This value lies in the achievable range of the setup in combination with the sample, which is illustrated by the shaded area in Panel (iv).

Subsequently, we measure the penetration depth when exciting the beads through the photonic waveguide. Figure 3c shows the measured FITC‐excitation in the evanescent field. Since the evanescent field is restricted to the waveguide surface, stronger localized spatial resolution is achieved. Hence, only one bead is visible in Panel (i) although several beads are present in the FOV (compare Figure S4 Supporting Information). The magnification of the single bead in Panel (ii) shows that the evanescently excited region of the bead is slightly elliptical. The reason for this shape is that the bead is located on the edge of the waveguide so that the evanescent field is spatially restricted in the *x*‐direction. The exponential fit of the detected fluorescence signal gives a penetration depth of 57 nm in water. This result is in good agreement with the corresponding simulation for the fundamental TE‐like mode of the used waveguide geometry as shown in Panel (iv).

In both experiments shown in Panel (b, iii) and (c, iii), the exposure time is 100 ms and the maximum of counts in the center of the bead, i.e., directly at the surface of the sample, is ≈2.5e3. This implies, that the power density at the surface in both experiments is almost equal. While the power exiting the microscope objective was ≈10 mW, the power exiting the fiber was only a tenth of that (≈1.2 mW). This again shows that significantly lower power is needed to arrive at the same power density at specific spots on the sample when a waveguide is used in comparison to objective exposure.

## 5 Evanescent Field Interaction with Living Neurons

**Figure 4:**
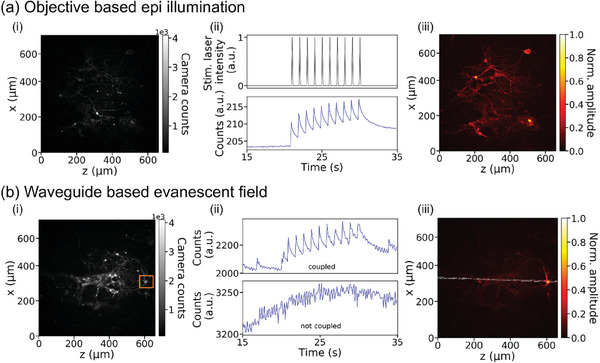
Demonstration of evanescent field interaction with living neurons. a) Fluorescence microscopy image of neurons growing on a photonic chip, Panel i). Neurons express mCherry‐labelled ChR‐2 and are incubated with the calcium indicator Rhod‐3, both visible owing to excitation with 561 nm. ii) Stimulation protocol for both objective and waveguide stimulation (upper plot). After 20 s background recording, the neurons are stimulated with 10 stimulation pulses with a frequency of 1 Hz and a duration of 60 ms. The lower plot shows the monitored time domain response of neurons averaged over the entire field of view based on Rhod‐3 fluorescence signal detection with a sampling frequency of 10 Hz and a camera exposure time of 10 ms. The causal relationship between stimulation and neural activity is clearly visible. For a more detailed description of the measurement protocol refer to the SI. iii) Normalized response of neurons shown in (i). The time signal in the relevant time window from 21 to 31 s is extracted for each pixel separately. In order to visualize cell response in the noisy signal, the time signal was transformed to the frequency domain using the fast Fourier transform (FFT). Amplitudes of integer multiples of 1 Hz (which corresponds to the stimulation frequency) were added up, normalized to the maximum value, and plotted for each pixel. Hence, bright pixels in (iii) show a coherent response to the stimulation pattern, while dark pixels do not respond. It is clearly visible that cells distributed over the entire field of view were stimulated. b) Fluorescence microscopy image of neurons, Panel (i). The upper plot of Panel (ii) shows the monitored time domain response of neurons averaged over the in (i) in an orange outlined rectangular area. The time domain signal peaks in coherence with the stimulation pulses (plotted in Figure 4a, Panel (ii)). The lower plot shows the recorded averaged time signal for the same region, however, for this measurement, the fiber was slightly misaligned so that no light was coupled into the waveguide. No response is visible. (iii) Normalized response of neurons shown in (i). The almost horizontally aligned stimulation waveguide is indicated in white. Only neurons in close vicinity to the waveguide show activity in the stimulation time frame, while neurons further away do not respond. Thus, the spatial resolution for stimulation is considerably improved.

The setup shown in Figure 3a is also used for in‐vitro experiments, in which the outreaching evanescent field of the waveguide interacts with living hippocampal neurons growing on the functionalized surface of the photonic platform. The chip design comprises multiple parallel, 4.5 µm wide and 300 nm high, straight OrmoComp waveguides with a spacing of 15.5 µm. Ta_2_O_5_ gratings underneath span the entire length of the sample so that each waveguide can be accessed individually with the cleaved fiber from above. The optogenetically engineered neurons growing on the surface of the waveguides express mCherry‐labeled ChR‐2 and are incubated with the calcium indicator Rhod‐3 for neural activity recording. Both fluorescent markers are visible in the fluorescence microscopy images in Panel (i) of both **Figure** **4**a,b, owing to excitation with 561 nm. To distinguish between transduced neurons and neurons, which are only incubated with Rhod‐3, an untagged version of ChR2 or a different calcium indicator such as Fura‐2 with excitation in the ultraviolet could be used.

Two different experiments are carried out in which the method to guide the stimulation light to the target cells is varied. In a reference measurement, both the stimulation laser beam to trigger ChR‐2 (488 nm) and the excitation laser beam to excite Rhod‐3 for imaging (561 nm) are focused by the 20x microscope objective on the sample surface in epi‐mode (Figure 4a). In the second experiment, the stimulation laser (491 nm) is coupled into the waveguide via the grating, while the excitation laser beam (561 nm) is still focused onto the sample by the objective (Figure 4b). Neural activity, i.e., a change in calcium concentration indicated by a varying Rhod‐3 fluorescence signal, is recorded through the objective underneath the chip in both cases.

The stimulation protocol for both measurements is shown in the upper plot of Figure 4a, Panel (ii). Background activity and noise are recorded for 20 s, followed by 10 stimulation pulses with a frequency of 1 Hz and a duration of 60 ms. The entire recording lasts 60 s. The lower plot of Panel (ii) shows the monitored time domain response of neurons averaged over the entire FOV (1200×1200 pixels) with a sampling frequency of 10 Hz and a camera exposure time of 10 ms. Neural spikes in response to each stimulation pulse are clearly visible. In order to determine which neurons or even parts of neurons in the FOV exactly responded, each pixel is evaluated separately. The corresponding time signal of a single pixel is much noisier compared to the averaged signal plotted in Panel (ii). To extract weak spiking signals from the noisy data, only the time window from 21 to 31 s in which the stimulation occurs is taken into account. The time signal is transformed to the frequency domain using the fast Fourier transform (FFT). For an ideal signal with 10 consecutive exponentially decaying peaks of 1 Hz, we would expect distinct peaks for frequencies with integer multiples of 1 Hz in the frequency domain (compare Figure S7, Supporting Information). Thus, as a figure of merit for a responding pixel, the amplitudes of 1, 2, 3, 4, and 5 Hz are added up and normalized to the pixel with the maximum value in the entire FOV. In this way, the direct current (DC) offset in the time domain is omitted. The normalized response of the neurons shown in Panel (i) is plotted in Panel (iii). Bright pixels show a coherent response to the stimulation pattern, while dark pixels do not respond. From this image, it is clearly visible that more or less all cells distributed over the whole FOV are stimulated. This is in line with our expectations since the beam diameter of the focused stimulation laser beam covers the entire region.

In the second experiment, the stimulation laser is coupled into the waveguide, which leads to spatially confined optical power (compare Figure S5, Supporting Information). The position of the waveguide is indicated in white in Figure 4b, Panel (iii). It is almost horizontally aligned and covers only a fraction of the FOV. The time domain signal averaged over the orange outlined rectangle illustrated in Figure 4b, Panel (i) is plotted in Panel (ii). Clear peaks in coherence with the stimulation pulses can be identified. The normalized amplitudes of integer multiples of 1 Hz are plotted in Panel (iii). Only neurons in close vicinity to the waveguide show activity in the stimulation time window, while neurons further away do not respond. Thus, the spatial resolution for neural stimulation is considerably improved compared to the stimulation via the objective. The lower plot of Panel (ii) shows the recorded averaged time signal for the same rectangular region. However, for this measurement, the fiber was slightly misaligned so that no light was coupled into the waveguide. In this plot, no peaks are visible, which hints that the cells do not respond. For a more detailed description of the measurement protocol and the evaluation procedure refer to the SI.

## 6 Discussion

Our low‐loss photonic platform at 488 nm wavelength with efficient fiber‐to‐chip coupling serves as a nontoxic substrate for neural cell culture, which we demonstrate in the experiment through the interaction of the waveguide's evanescent field with living neurons. A clear cell activity coherent with the stimulation pattern through the waveguide is observed.

The platform offers high stimulation resolution in the out‐of‐plane (*y*‐) direction on the nanoscale. This is verified by determining the penetration depth of the evanescent field, which amounts to ≈57 nm in water. In contrast, epi‐illumination with a microscope objective leads to light penetrating the entire sample volume in the *y*‐direction, so that the stimulation resolution cannot be defined. This unrestricted illumination is helpful in experiments, in which also ChR‐2 located in higher cell layers are supposed to be triggered. In the in‐plane (*x,z*‐) direction, the stimulation resolution of the objective‐based stimulation is determined by the diameter of the focused laser beam, which is larger than the FOV of the camera. This leads to a large‐area exposure as visible in Figure 4a; Figure S4, Supporting Information) and hence precise stimulation of only specific cells (or beads) in the FOV is challenging. The result is a large heat input into the sample, a widespread bleaching of fluorescent dyes, and unnecessary stress of off‐target cells.

A key advantage of the presented photonic platform compared to conventional approaches is the separation of the stimulation and imaging pathway. The stimulation pathway via waveguide enables very specific local stimulation of individual cells in the in‐plane (*x,z*‐) direction with high resolution as shown in Figure 4b. This is due to the confinement of the light in the waveguide (see Figure S5, Supporting Information), which also limits the sample volume subjected to heat input and bleaching. At the same time, the neural activity in a larger FOV can be imaged independently. The *x,z*‐stimulation resolution is determined by the design of the photonic circuit, the exact overlap between the stimulation waveguides and the neurons, and the control of individual waveguides. In general, the stimulation resolution can be improved by minimizing the overlap of the cell with the individual stimulation spots or by decreasing the waveguide dimensions. The cut‐off width of an OrmoClear waveguide is ≈350 nm for a wavelength of 488 nm.

In this work, the photonic design consists of parallel waveguides of which only one is selected at a time. An overlap of a neuron with multiple waveguides allows for the simultaneous or sequential selection of several waveguides and, thus, enables flexible spatial and temporal stimulation patterns in one direction (here *x*‐direction). This layout even allows for experiments in which part of a cell is stimulated with a certain wavelength, while another part is inhibited by another wavelength in an adjacent waveguide given that the cell expresses the respective light‐triggered proteins. A smaller pitch between adjacent waveguides increases the number of individually addressable waveguides per area and, thus, the stimulation resolution.

Flexible spatial and temporal stimulation configurations in 2D (here *x*‐ and *z*‐direction) are achieved with an array of waveguide crossings, for instance. The guided optical power in the waveguides can be adjusted so that the threshold power density for cell stimulation is only exceeded at the crossing points, where the power of two or more waveguides adds up.^[^ ^49^ ^]^ In this way, we can restrict the spatial stimulation patterns to areas with a size of a few squared micrometers or even nanometres, which we can individually address. This allows for the stimulation of single neurons or even single branches of neurons. A side benefit of the reduced power is the reduction of phototoxicity.

However, the waveguides are unsuitable for large‐area stimulation as it is possible with the microscope objective. In order to stimulate an entire cell, the total overlap of waveguide and cell can be increased by using meander‐shaped designs, for instance. In this way, very long interaction ranges are possible and larger portions of a cell can be stimulated with the same waveguide. This is advantageous compared to the out‐coupling method presented by Welkenhuysen and coworkers.^[^ ^23^ ^]^ We also note that the fact that neurons in close vicinity to a waveguide surface can be stimulated by the evanescent field should also be taken into account in experiments using invasive optoprobes, where it might lead to unwanted cross‐talk.

Independently from the stimulation pathway, the high spatial resolution read out even of single synapses is accomplished through the 100× magnification oil immersion objective of the fluorescence microscope setup. The signal‐to‐noise ratio and resolution in the *z*‐direction (here defined as the *y*‐direction) are additionally improved if the microscope is used in TIRF mode. At the same time, our approach further enables capturing a large FOV with a 20× magnification (or less) objective. This is advantageous for the study of large‐scale neural network dynamics. The recording from underneath requires a transparent substrate which at first sight is unusual for photonic circuits. However, the used hybrid polymer Ormocer can be readily spin‐coated on a standard microscope cover glass serving as an inexpensive substrate. The developed NIL process to pattern low‐loss Ormocer waveguides is an easy and cost‐effective replication technique suitable for mass production. It can be carried out with a multiple‐use stamp even at wafer scale and without the need for expensive lithography equipment.

An additional benefit is that due to the high confinement in the waveguide only an extremely low power is necessary in order to exceed the triggering threshold of ChR‐2. This reduces the heat input into cell tissue and, in addition, avoids phototoxicity compared to stimulation via objective, by which a large amount of cells is simultaneously illuminated.

Having said this, there is still room for improvement and further investigations. An important aspect is cross talk which might originate from the optical fiber but also from the photonic waveguide. In the first case, the distance between the fiber and the cells under investigation can be increased with the help of sufficiently long waveguides. Thus, scattered light that is not coupled into the waveguide cannot lead to unintended cell stimulation. The minimum distance should be evaluated in both simulation and experiment. With regard to the waveguide, several issues have to be taken into account. First, the distance between neighboring waveguides has to be sufficiently large so that no directional coupling occurs. This limits the spatial resolution of two independent stimulation spots. Second, a rough surface of the waveguide leads to the coupling of guided modes to radiation modes. In this case, the optical power is not confined to the waveguide anymore but can have any propagation direction. This can be fixed by reducing the surface roughness of the waveguide. Radiation loss can also occur due to bent waveguides, which is not negligible in particular for the used low‐index contrast system.^[^ ^50^ ^]^ A systematic investigation is necessary to determine the minimum bend radius at which the radiated power does not stimulate off‐target cells.

In our experiments, the excitation pathway for imaging Rhod‐3 is through the objective microscope. However, with the photonic chip, it is also possible to implement waveguide‐based TIRF imaging.^[^ ^51^ ^]^ This means, that in addition to the stimulation wavelength, also the excitation wavelength is coupled into the same or a close waveguide. This is especially advantageous if a large FOV is needed but a high *z*‐resolution has to be maintained.^[^ ^36^ ^]^

For aligned positioning of cells with respect to the photonic circuitry, dip‐pen nanolithography can be employed. This process allows for accurate surface functionalization at specific spots on the sample, which restricts the areas of cell growth. In this way, the neuron network can be adapted to the photonic circuit for specialized investigations, for example, the stimulation of single neurons. Moreover, by the choice of the used opsin or specific targeting motifs to restrict opsin appearance to certain cell segments even more possibilities of exploration emerge and enrich our tool for precise investigation of neurons on the synapse level as well as complete network dynamics.

## 7 Conclusion

In summary, we demonstrate a low‐loss photonic platform with a waveguide propagation loss of –0.9 ± 0.2 dB cm^−1^ at 489 nm wavelength. Convenient fiber‐to‐chip access to on‐chip waveguides compatible with NIL fabrication is realized using high‐index etched Ta_2_O_5_ or Al gratings. We experimentally determined the evanescent field penetration depth of the waveguides in an aqueous environment to be 57 nm with the help of FITC‐covered microbeads, which is in good agreement with our simulation. We further show that the presented photonic platform is nontoxic for living neural cell culture and uses surface functionalization to induce neural growth. Distinct neural activity can be observed in coherence with the stimulation pattern through the photonic waveguide. We expect that the versatility of our photonic platform for evanescent field stimulation of in‐vitro neurons makes it a highly attractive alternative to common measurement procedures and that it will positively contribute to further progress in neuroscience research.

## 8 Experimental Section

### Waveguide Fabrication

The waveguides were patterned via nanoimprint lithography (NIL). Ormocer was spin‐coated on a preprocessed substrate, brought into contact with a transparent stamp, and cured via UV exposure. After detachment, a thermal treatment was carried out to increase the adhesion of the structures to the substrate. The residual layer of the final sample, which is characteristic of the NIL process,^[^ ^52^ ^]^ was minimized to 40 nm thickness to ensure good mode confinement in the waveguide. This was achieved by diluting the Ormocer resist, hence, enabling a thin initial resist thickness of 160 nm after spin coating. The used imprint stamp, comprising the negative image of the pattern, was beforehand fabricated with the same imprint process. For this purpose, a master chip was used featuring a 330 nm thick structured SiN layer (realized by electron‐beam lithography and dry etching), which also defineed the height of the imprinted waveguides. After photo‐curing and hardbake, the volume of the Ormocer resists shrank so that a mean waveguide height of 300 nm resulted. More detailed process parameters can be found in the SI.

### Edge‐Coupling Access to On‐Chip Waveguides

The refractive index contrast between the OrmoClear waveguide with *n*_OrmoClear  =  1.566 @ *λ*  =  491 nm (OrmoComp waveguide with *n*_OrmoComp  =  1.527 @ *λ*  =  491 nm) and the water cladding (*n*_water  =  1.340 @ *λ*  =  491 nm) amounted 0.144 (0.122). Thus, the coupling strength of a common Ormocer grating coupler was too low for efficient fiber‐to‐chip coupling with single‐mode fiber.^[^ ^53^, ^54^ ^]^ In order to maximize the coupling efficiency by edge‐coupling instead, the waveguide width was chosen to match the mode field diameter of the used optical fiber, assuming that the beam exiting the fiber has the shape of a Gaussian beam with a beam waist diameter equal the mode field diameter. The overlap integral between this beam and the fundamental TE‐like mode of a 4.5 µm wide and 340 nm high waveguide (slab height included) was calculated for various distances in *z* and optimal alignment in both *x*‐ and *y*‐direction and plotted in Figure S2 (Supporting Information). If the fiber and waveguide facet (SEM image shown in the inset) were nearly in contact (*z* = 0 µm), the coupling efficiency amounted –3.6 dB. For a distance of *z*  =  10 µm, the efficiency was already reduced to –4.2 dB and then decreases almost linearly with increasing distance. Fresnel reflection at the interfaces was negligible and not considered in this calculation. In order to facilitate the cleaving of the waveguide facet, the devices were stamped on the thermal oxide layer of a silicon substrate. For five different 2 mm long, s‐bend waveguides, an experimental coupling efficiency of – 5.5 ± 0.3 dB/facet was achieved (propagation losses neglected). This was a reasonable value compared to the calculation. The edge‐coupling method was used to determine waveguide propagation losses.

### Primary Mouse Hippocampal Neuron Culture Preparation

Hippocampi were excised from new‐born pups (C57/BL6 mice) and incubated in 0.1% trypsin (Sigma T1005) solution with 20 µg mL^−1^ DNase I (Deoxyribonuclease I, Serva 18 535) for 30 min at 37 °C. After washing using plating medium (2.5 g glucose, 100 mg NaHCO3, 50 mg transferrin (Calbiochem), 10% FBS (Biochrome), 1 mL 0.2 ML‐glutamine (Sigma), 2.5 mg insulin (Sigma) in 500 mL MEM (Gibco)), the cells were dissociated by trituration in 1 mL of plating medium with 40 µg mL^−1^ DNase I. The cells were counted and plated on Poly‐D‐Lysin‐coated coverslips with the approximate density of 20 000 cells cm^−2^ in growth medium (Neurobasal A medium (Gibco), 2% B27 (Gibco), 2 mm Glutamax (Gibco), 10 units mL penicillin and 10 µg mL streptomycin (Gibco)). The glass coverslips were previously coated by incubation in 0.75 µg Poly‐D‐Lysin solution for 1 h at 37 °C. To prevent excessive astrocytes proliferation, 2 days after the plating Ara‐C (Citarabin, Sigma C6645) was added to the medium of growing cells to a final concentration of 4 µm. Lentiviral particles for ChR2 transduction were added 3 days after plating at a concentration of 1 µL mL^−1^.

### Lentiviral Particle Preparation

HEK293FT cells were transfected 24 h after plating or upon reaching ≈70% confluency on 10 mm round culture plates. They were transfected by TransiT‐293 transfection reagent (Mirus). Per 10 mm plate, 23.6 µL TransiT‐293 reagent is added to 1.18 mL Opti‐MEM media (Gibco). This mixture is incubated at RT for 10 min. After incubation 5 µg lentiviral construct (pLenti‐CaMKIIa‐hChR2(H134R)‐mCherry‐WPRE), 2.5 µg helper plasmid, viral envelope (ENV, pMD2.G) and 2.5 µg of another packaging plasmid (pCMV R8.2) were added to Opti‐MEM and TransiT reagent mixture. This is followed by quick vortexing and incubation for 20 min at RT. Cell medium was exchanged with fresh medium and a complete mixture of the DNA/TransiT reagent containing Opti‐MEM medium was added to each 10 mm plate. Then, the plates were transferred into a humidified incubator (37 °C, 5% CO2) for 72 h. All DNA plasmids used for transfection were purified with an endotoxin‐free maxiprep purification kit (Qiagen). After 72 h, the medium was collected from 4–6 10 mm plates for harvesting lentiviral particles. The media were filtered through the 0.4‐micron filter to remove cell debris. Afterward, media were centrifuged at 25000 RPM for 90 min. The supernatant was removed, and the pellet was resuspended in 100–200 µL Dulbecco's phosphate‐buffered saline (DPBS, Gibco), and aliquots were stored at −80 ˚C, for transduction of neurons.

## Conflict of Interest

The authors declare no conflict of interest.

## Supporting information

Supporting Information

## Data Availability

The data that support the findings of this study are available from the corresponding author upon reasonable request.;

## References

[1] K. Deisseroth , Nat. Methods 2011, 8, 26. 21191368 10.1038/nmeth.f.324 PMC6814250

[2] Y. Sun , M. Li , S. Cao , Y. Xu , P. Wu , S. Xu , Q. Pan , Y. Guo , Y. Ye , Z. Wang , H. Dai , X. Xie , X. Chen , W. Liang , Int. J. Mol. Sci. 2022, 23, 1800. 35163726 10.3390/ijms23031800 PMC8836693

[3] E. S. Boyden , F. Zhang , E. Bamberg , G. Nagel , K. Deisseroth , Nat. Neurosci. 2005, 8, 1263. 16116447 10.1038/nn1525

[4] G. Nagel , T. Szellas , W. Huhn , S. Kateriya , N. Adeishvili , P. Berthold , D. Ollig , P. Hegemann , E. Bamberg , Proc. Natl. Acad. Sci. USA 2003, 100, 13940. 14615590 10.1073/pnas.1936192100 PMC283525

[5] F. Zhang , L.‐P. Wang , M. Brauner , J. F. Liewald , K. Kay , N. Watzke , P. G. Wood , E. Bamberg , G. Nagel , A. Gottschalk , K. Deisseroth , Nature 2007, 446, 633. 17410168 10.1038/nature05744

[6] A. R. Mardinly , I. A. Oldenburg , N. C. Pégard , S. Sridharan , E. H. Lyall , K. Chesnov , S. G. Brohawn , L. Waller , H. Adesnik , Nat. Neurosci. 2018, 21, 881. 29713079 10.1038/s41593-018-0139-8 PMC5970968

[7] J. Deubner , P. Coulon , I. Diester , Curr. Opin. Struct. Biol. 2019, 57, 157. 31082625 10.1016/j.sbi.2019.04.003

[8] P. Schoenenberger , Y.‐P. Z. Schärer , T. G. Oertner , Exp. Physiol. 2010, 96, 34. 20562296 10.1113/expphysiol.2009.051219

[9] A. R. Adamantidis , F. Zhang , A. M. Aravanis , K. Deisseroth , L. De Lecea , Nature 2007, 450, 420. 17943086 10.1038/nature06310 PMC6744371

[10] F. Albers , L. Wachsmuth , T. M. Van Alst , C. Faber , Mol. Imaging Biol. 2018, 20, 171. 29027094 10.1007/s11307-017-1130-6

[11] B. R. Arenkiel , J. Peca , I. G. Davison , C. Feliciano , K. Deisseroth , G. J. Augustine , M. D. Ehlers , G. Feng , Neuron 2007, 54, 205. 17442243 10.1016/j.neuron.2007.03.005 PMC3634585

[12] B. Li , K. Lee , S. C. Masmanidis , M. Li , J. Neural Eng. 2018, 15, 046008. 29629879 10.1088/1741-2552/aabc94 PMC6021216

[13] E. Segev , J. Reimer , L. C. Moreaux , T. M. Fowler , D. Chi , W. D. Sacher , M. Lo , K. Deisseroth , A. S. Tolias , A. Faraon , M. L. Roukes , Neurophotonics 2016, 4, 1. 10.1117/1.NPh.4.1.011002 PMC5136672 27990451

[14] R. Scharf , T. Tsunematsu , N. Mcalinden , M. D. Dawson , S. Sakata , K. Mathieson , Sci. Rep. 2016, 6, 28381. 27334849 10.1038/srep28381 PMC4917834

[15] V. Lanzio , G. Telian , A. Koshelev , P. Micheletti , G. Presti , E. D'arpa , P. De Martino , M. Lorenzon , P. Denes , M. West , S. Sassolini , S. Dhuey , H. Adesnik , S. Cabrini , Microsystems Nanoeng 2021, 7, 40. 10.1038/s41378-021-00263-0 PMC8433201 34567754

[16] N. Broguiere , I. Lüchtefeld , L. Trachsel , D. Mazunin , R. Rizzo , J. W. Bode , M. P. Lutolf , M. Zenobi‐Wong , Adv. Mater. 2020, 32, 1908299. 10.1002/adma.201908299 32390195

[17] O. A. Shemesh , D. Tanese , V. Zampini , C. Linghu , K. Piatkevich , E. Ronzitti , E. Papagiakoumou , E. S. Boyden , V. Emiliani , Nat. Neurosci. 2018, 21, 896. 10.1038/s41593-018-0097-1 29549318

[18] M. Dal Maschio , F. Difato , R. Beltramo , A. Blau , F. Benfenati , T. Fellin , Opt. Express 2010, 18, 18720. 20940765 10.1364/OE.18.018720

[19] G. Katona , G. Szalay , P. Maák , A. Kaszás , M. Veress , D. Hillier , B. Chiovini , E. S. Vizi , B. Roska , B. Rózsa , Nat. Methods 2012, 9, 201. 22231641 10.1038/nmeth.1851

[20] K. M. N. S. Nadella , H. Ros , C. Baragli , V. A. Griffiths , G. Konstantinou , T. Koimtzis , G. J. Evans , P. A. Kirkby , R. A. Silver , Nat. Methods 2016, 13, 1001. 27749836 10.1038/nmeth.4033 PMC5769813

[21] H. Adesnik , L. Abdeladim , Nat. Neurosci. 2021, 24, 1356. 34400843 10.1038/s41593-021-00902-9 PMC9793863

[22] E. Ronzitti , V. Emiliani , E. Papagiakoumou , Front. Cell. Neurosci. 2018, 12, 469. 30618626 10.3389/fncel.2018.00469 PMC6304748

[23] M. Welkenhuysen , L. Hoffman , Z. Luo , A. De Proft , C. Van Den Haute , V. Baekelandt , Z. Debyser , G. Gielen , R. Puers , D. Braeken , Sci. Rep. 2016, 6, 20353. 26832455 10.1038/srep20353 PMC4735812

[24] A. Steude , E. C. Witts , G. B. Miles , M. C. Gather , Sci. Adv. 2016, 2, e1600061. 27386540 10.1126/sciadv.1600061 PMC4928914

[25] S. Weigel , T. Osterwalder , U. Tobler , L. Yao , M. Wiesli , T. Lehnert , A. Pandit , A. Bruinink , PLoS One 2012, 7, e50714. 23251379 10.1371/journal.pone.0050714 PMC3520951

[26] D. Axelrod , T. P. Burghardt , N. L. Thompson , Ann. Rev. Biophys. Bioeng. 1984, 13, 247. 6378070 10.1146/annurev.bb.13.060184.001335

[27] F. Zhang , L.‐P. Wang , E. S. Boyden , K. Deisseroth , Nat. Methods 2006, 3, 785. 16990810 10.1038/nmeth936

[28] C. Kaspar , J. Lehrich , A. Ivanenko , J. Klingauf , W. H. P. Pernice , In Proc. SPIE 11227, Optogenetics Opt Manip, SPIE, Bellingham, WA 2020, 11227, 1122703.

[29] A. Minta , J. P. Y. Kao , R. Y. Tsien , J. Biol. Chem. 1989, 264, 8171. 2498308

[30] J. T. Russell , Br. J. Pharmacol. 2011, 163, 1605. 20718728 10.1111/j.1476-5381.2010.00988.x PMC3166690

[31] D. Kohler , G. Schindler , L. Hahn , J. Milvich , A. Hofmann , K. Länge , W. Freude , C. Koos , Light Sci. Appl. 2021, 10, 64. 33767136 10.1038/s41377-021-00486-w PMC7994412

[32] W. D. Sacher , X. Luo , Y. Yang , F.‐D. Chen , T. Lordello , J. C. C. Mak , X. Liu , T. Hu , T. Xue , P. Guo‐Qiang Lo , M. L. Roukes , J. K. S. Poon , Opt. Express 2019, 27, 37400. 31878521 10.1364/OE.27.037400 PMC7046040

[33] S. Romero‐García , F. Merget , F. Zhong , H. Finkelstein , J. Witzens , Opt. Express 2013, 21, 14036. 23787593 10.1364/OE.21.014036

[34] P. Muñoz , G. Micó , L. Bru , D. Pastor , D. Pérez , J. Doménech , J. Fernández , R. Baños , B. Gargallo , R. Alemany , A. Sánchez , J. Cirera , R. Mas , C. Domínguez , Sensors 2017, 17, 2088. 28895906 10.3390/s17092088 PMC5620990

[35] A. Z. Subramanian , P. Neutens , A. Dhakal , R. Jansen , T. Claes , X. Rottenberg , F. Peyskens , S. Selvaraja , P. Helin , B. Dubois , K. Leyssens , S. Severi , P. Deshpande , R. Baets , P. Van Dorpe , IEEE Photonics J 2013, 5, 2202809.

[36] A. Priyadarshi , F. T. Dullo , D. L. Wolfson , A. Ahmad , N. Jayakumar , V. Dubey , J.‐C. Tinguely , B. S. Ahluwalia , G. S. Murugan , Commun. Mater. 2021, 2, 85.

[37] L. Liebermeister , N. Heinrichs , B. Florian , M. Zeitlmair , T. Tashima , (Preprint) arXiv:1710.03095 [quant‐ph] 2017.

[38] D. J. Blumenthal , APL Photonics 2020, 5, 020903.

[39] M. Herder , J. J. Klein , M. Vogler , M.‐M. Russew , A. Schleunitz , G. Grützner , In 3D Print. Opt. Components, 2021, (Ed.: A. Heinrich ), Springer Nature, Switzerland AG, pp. 263–297.

[40] F. P. Payne , J. P. R. Lacey , Opt. Quantum Electron. 1994, 26, 977.

[41] A. Ovsianikov , S. Schlie , A. Ngezahayo , A. Haverich , B. N. Chichkov , J. Tissue Eng. Regener. Med. 2007, 1, 443. 10.1002/term.57 18265416

[42] A. Doraiswamy , A. Ovsianikov , S. Gittard , N. Monteiro‐Riviere , R. Crombez , E. Montalvo , W. Shen , B. Chichkov , R. Narayan , J. Nanosci. Nanotechnol. 2010, 10, 6305. 21137723 10.1166/jnn.2010.2636

[43] S.‐H. Yoon , Y. K. Kim , E. D. Han , Y.‐H. Seo , B. H. Kim , M. R. K. Mofrad , Lab Chip 2012, 12, 2391. 22534829 10.1039/c2lc40084g

[44] F. Milos , A. Belu , D. Mayer , V. Maybeck , A. Offenhäusser , Adv. Biol. 2021, 5, 2000248.

[45] D. J. Lorang , D. Tanaka , C. M. Spadaccini , K. A. Rose , N. J. Cherepy , J. A. Lewis , Adv. Mater. 2011, 23, 5055. 21989713 10.1002/adma.201102411

[46] J. Hiltunen , A. Kokkonen , J. Puustinen , M. Hiltunen , J. Lappalainen , IEEE Photonics Technol. Lett. 2013, 25, 996.

[47] D. Loerke , Molecular Dynamics of Clathrin Proteins at Endocytic Sites Studied with Evanescent‐Wave Microscopy, Georg‐August‐Universität zu Göttingen, Göttingen, Germany, 2004.

[48] A. L. Mattheyses , D. Axelrod , J. Biomed. Opt. 2006, 11, 014006. 16526883 10.1117/1.2161018

[49] J. Feldmann , N. Youngblood , C. D. Wright , H. Bhaskaran , W. H. P. Pernice , Nature 2019, 569, 208. 31068721 10.1038/s41586-019-1157-8 PMC6522354

[50] L. Li , G. Nordin , J. English , J. Jiang , Opt. Express 2003, 11, 282. 19461734 10.1364/oe.11.000282

[51] R. Diekmann , Ø. I. Helle , C. I. Øie , P. Mccourt , T. R. Huser , M. Schüttpelz , B. S. Ahluwalia , Nat. Photonics 2017, 11, 322.

[52] T. Balla , S. M. Spearing , A. Monk , J. Phys. D. Appl. Phys. 2008, 41, 174001.

[53] R. Bruck , R. Hainberger , Appl. Opt. 2010, 49, 1972. 20357883 10.1364/AO.49.001972

[54] L. Wang , Y. Li , M. Garcia Porcel , D. Vermeulen , X. Han , J. Wang , X. Jian , R. Baets , M. Zhao , G. Morthier , J. Appl. Phys. 2012, 111, 114507.

