# Supplementary material for: Biohybrid Photonic Platform for Subcellular Stimulation and Readout of In Vitro Neurons
Source: Adv Sci (Weinh). 2024 Jan 2;11(12):2304561. doi: 10.1002/advs.202304561 (PMC10966564; doi:10.1002/advs.202304561)
Supplement: Supplementary file 1 — Supporting Information [file ADVS-11-2304561-s001.pdf]

ADVANCED SCIENCE
Open Access

Supporting Information

for *Adv. Sci.*, DOI 10.1002/advs.202304561

Biohybrid Photonic Platform for Subcellular Stimulation and Readout of In Vitro Neurons

*Corinna Kaspar, Alexander Ivanenko, Julia Lehrich, Jürgen Klingauf and Wolfram H.P. Pernice**

Supporting Information

Biohybrid photonic platform for sub-cellular stimulation and readout of in-vitro neurons

Corinna Kaspar, Alexander Ivanenko, Julia Lehrich, Jürgen Klingauf, Wolfram H.P. Pernice

Power density at the waveguide surface

Outside of the waveguide, the evanescent power density decays exponentially according to

$$I(z) = I_{surf}\ \exp(\frac{-y}{d_p})\ , \qquad \text{(Eq. 1)}$$

for the area above the waveguide, where I_{surf} is the power density at the surface of the waveguide and d_p is the penetration depth, i.e. the distance at which the intensity has declined to 1/e of its maximum value. The penetration depth d_p can be expressed by

$$d_p = \frac{\lambda}{4\pi\sqrt{n_1^2 \sin^2 \vartheta - n_2^2}}\ , \qquad \text{(Eq. 2)}$$

where λ is the wavelength and n_1 and n_2 the refractive index of the core and cladding, respectively, and ϑ the incident angle. The absolute power density at the level of the waveguide surface I_{surf} is plotted in Figure S1 for the first three TE-like modes, where the input power is set to 1 mW. Since the waveguide has dimensions on the nm to µm scale the optical mode is tightly confined and very high power densities are achieved with moderate input power. The computed power densities peak at about 3.3e5 mW/mm^2 for TE00, TE10 and TE20.

Theoretical efficiency of edge-coupling approach

The overlap integral between a Gaussian beam with a mode field diameter equal to the mode field diameter of a single mode fiber at 488 nm and the fundamental TE-like mode of a 4.5 µm wide and 340 nm high waveguide (slab height included) is calculated for various distances in z and optimal alignment in both *x*- and *y*-direction and plotted in Figure S2. If the fibre and waveguide facet (SEM image shown in the inset) are nearly in contact (z ≈ 0 µm), the coupling efficiency amounts - 3.6 dB. For a distance of *z* = 10 µm, the efficiency is already reduced to - 4.2 dB and then decreases almost linearly with increasing distance. Fresnel reflection at the interfaces is negligible and not considered in this calculation.

Photonic device fabrication

The photonic waveguides are fabricated via nanoimprint lithography (NIL) using the SmartNIL technology of a mask alignment system EVG620 NT (EV Group, Austria). For the NIL process, a stamp with the negative image is needed, which is casted from a master chip featuring the same pattern as the final sample.

Master chip fabrication

The master chip is fabricated from a 330 nm thick silicon nitride (SiN) layer on silicon oxide (3300 nm) on a silicon wafer (Rogue Valley Microdevices, Oregon) using electron-beam lithography (EBPG 5150, Raith, Germany). A 300 nm thick layer of the negative tone resist AR-N 7520.12 (Allresist, Germany) is spin coated on the 20 mm x 20 mm big chip, prebaked for 60 s at 85 °C on a hotplate and exposed. The resist is developed in MF-319 (micro resist technology, Germany) for 75 s and a hard bake is carried out at 85 °C for 60 s. The pattern is transferred into the SiN layer via reactive ion etching (Oxford PlasmaPro 80, Oxford Instruments) using a CHF_3/O_2 plasma and resist residuals are removed by a following O_2 plasma.

Imprint stamp fabrication

The stamp is fabricated with the same imprint process as the final sample. For this, a sticky, transparent carrier foil (EV Group) is inserted in the SmartNIL tool. The master chip is spin coated with an anti-sticking layer (EVGASL 1, EV Group) and baked for 5 min at 120 °C. Afterwards, the

working stamp material UV/AS1 (EV Group) is spin coated. Master chip and carrier foil are brought into contact and the working stamp material is cured via UV-exposure through the transparent foil. Afterwards, chip and foil are separated again and the structured working stamp, featuring the negative image of the master stamp, is attached to the carrier foil.

Sample – grating fabrication

As substrate either a 500 µm thick fused silica wafer with a 200 nm thick deposited tantalum pentoxide (Ta_2O_5) layer diced to 15 mm x 15 mm, (Siegert Wafer, Germany) or a 130 µm thick microscope cover glass with a diameter of 18 mm (Assistant, Germany) is used.

In the first case, the Ta_2O_5 layer is structured via electron-beam lithography. To avoid charging during the exposure, a 10 nm thick aluminum (Al) layer is deposited. As adhesion promoter TiPrime (micro resist technology) is used and baked for 2 min at 120 °C. A 330 nm thick layer of the negative tone resist ma-N2403 (micro resist technology) is spin coated and prebaked at 90 °C for 2 min. At one edge of the sample, the resist is removed with MF-319 to lay open the Al, which is connected to the sample holder of the electron beam system with a copper strip to enable electrical discharge. After exposure, the resist is developed in a 1:3 mixture of deionized water and MF-319 for 120 s. At the end of this step, the Al between the structures is also etched away as soon as it is exposed to the alkaline developer. The pattern is then transferred into the Ta_2O_5 layer by RIE with a $CHF_3/CF_4/Ar$ plasma and the residual resist is stripped with a following O_2 plasma. The remaining aluminum, which was previously covered by the resist, is removed with MF-319.

On the microscope cover glass, a grating is etched in a 40 nm thick deposited Al layer (see AFM measurement in Figure S3). As etch mask serves a 330 nm thick ma-N 2403 layer, which is structured via electron-beam lithography as described above. The development time is 7 min to ensure a fully etched grating. The residual resist is stripped in acetone.

Sample – waveguide fabrication

The waveguide material is either OrmoComp®-500nm_XP or OrmoClear®FX-500nm_XP (micro resist technology). The relevant difference between both is the higher refractive index of OrmoClear (n_OrmoClear = 1.566 @ λ = 491 nm), while OrmoComp has a refractive index of

n_OrmoComp = 1.527 @ λ = 491 nm. To ensure functional waveguides on the microscope cover glass with a refractive index of $n_{\text{cover glass}}$ = 1.523 @ λ = 589 nm, OrmoClear is used. The substrate (independent on the material) is treated with an oxygen plasma and afterwards spin coated with the adhesion promoter OrmoPrime (micro resist technology), which is baked for 2 min at 80 °C. Ormocomp and Ormoclear are both diluted 1:1 with propylenglycolmonomethyletheracetat (PGMEA) to achieve a resist thickness of 160 nm via spin coating. A prebake of 2 min at 80 °C follows. The patterning of the resist is done via the NIL process described above. After exposure, a hard bake at 130°C for 10 min is carried out to improve the adhesion to the substrate.

With the used EVG620 NT system, no alignment between the structured gratings on the sample and the working stamp on the carrier foil is possible. Thus, the gratings are extended over the entire length of the chip to ensure an overlap.

Estimation of waveguide propagation loss due to scattering

The analytical model according to Payne and Lacey[40] relates loss induced by scattering to the surface roughness of the waveguide with width $2d$ according to

$$\alpha = \varphi^2(d)(n_1^2 - n_2^2)^2 \frac{k_0^3}{4\pi n_1} \cdot S\,, \qquad \text{(Eq.3)}$$

where φ is the normalised modal field at the core-cladding interface $y = \pm d$, n_1 and n_2 the refractive index of core and cladding, respectively, and k_0 the free space wavenumber. The parameter S depends on the free space wavenumber, the refractive index of the cladding, the modal propagation constant, the correlation length and the standard deviation of the surface roughness. The latter two are obtained by an exponential autocorrelation function of the waveguide's surface roughness.[40] Since the highest overlap of the mode is at the two core-cladding boundaries in *y*-direction (cf. Figure 1(b)), we assume that scattering only occurs at the surface, while the interface with the substrate is considered to be smooth. From the AFM scan in Figure S3, a correlation length of 44.4 ± 13.9 nm and a standard deviation of 2.7 ± 0.5 nm is extracted. Inserting all values in Equation 3 yields a scattering loss of α = 1.5 dB/cm. This value is in the same order of magnitude as the total measured loss.

Evanescent field interaction with fluorescent beads

Figure S4 shows two fluorescence microscopy images of FITC-covered microbeads (microParticles, Germany) excited in Epi-mode. (a) displays the same FOV as shown in Figure 3(b), Panel (i) in the main text. The same six beads are visible in both epi and TIRF mode. This is due to the fact that the diameter of the focused laser beam covers the entire FOV also in TIRF mode. In contrast, the waveguide based evanescent field is spatially confined. The FOV shown in (b) corresponds to the image section displayed in Figure 3(c), Panel (i) in the main text. While in the Epi-mode several beads are visible, only one bead in the entire FOV, which is situated directly on top of the waveguide, is excited by the evanescent field. Thus, the spatial resolution is substantially increased compared to the objective excitation.

The used silica beads have a refractive index of about 1.42. In an aqueous environment with refractive index of 1.34, this would lead to a propagating wave inside the bead when exposed to the evanescent field. As a consequence, the image would not show radial symmetric fluorescence but rather distorted spots due to scattering.[48] For this reason, the indices of beads and surrounding medium have to be matched, which is why an index-matched sucrose solution is used. In order to determine the penetration depth of the evanescent field, the detected camera counts of the TIRF image are plotted as a function of the distance to the sample surface. An exponential fit yields the value for the penetration depth in sucrose solution. From this value, the penetration depth for a water environment can be calculated with the formula [47]

$$d_{p,water} = \sqrt{\frac{1}{\frac{1}{d_{p,sucrose}^2}+\frac{16\pi^2}{\lambda^2}(n_{sucrose}^2-n_{water}^2)}}\,.$$

Stimulation waveguide

For the waveguide based evanescent field measurement shown in Figure 4(b) in the main text, the grating and thus also the fiber to couple light into the waveguide where about 1.4 mm away from the FOV (in positive *x*-direction). If we assume a cell height of 50 µm, light scattered from the fiber under an angle of less than 2 ° from the sample plane can contribute to cell stimulation. This is a very shallow angle, which lets us assume that the impact is little.

In Figure S5, the same FOV is displayed as in Figure 4(b). The stimulation laser (491 nm, ca. 4.1 mW) is coupled into the waveguide via the Ta_2O_5 grating, while the excitation laser (561 nm) is turned off.

The recorded signal is an overlap of scattering from the waveguide surface (due to roughness or index variations in the surrounding caused by cells or the medium in which they grow) and Rhod-3 fluorescence since its excitation tail reaches till 491 nm. The background noise is about 100 counts. In Figure S5(b), the counts are plotted along x for y = 660 µm. For *x* = 0 µm, i.e. in the upper left corner in (a), there is only background noise. With decreasing distance to the fiber (increasing *x*), gradually more counts are detected. A linear fit yields a slope of 1.4 counts per 100 µm. Figure S5(c) shows the same plot for *y* = 347 µm, i.e. a little above the waveguide. While the intercept is increased to 105 counts, the slope is the same as for (b). Thus, close to the waveguide, there is only a slight increase of intensity. In direction to the fiber, more scattering light (an increase of about 9 counts in the whole FOV) is detected. However, the increase is less than 1 % of the values detected on top of the waveguide. This makes us assume, that the scattering light from the fiber is negligible and the neurons were only stimulated by the evanescent field of the waveguide, which is in agreement with our observations in Figure 4(b).

Measurement protocol for neural cell stimulation

The measurement protocol is plotted in Figure S6. The whole measurement duration is 60 s. Camera exposure and imaging laser (561 nm) are synchronized. Every 100 ms (≙ 10 Hz), a fluorescence image of Rhod-3 signal is captured with an exposure time of 10 ms (blue line). The background noise and cell activity is recorded for 20.91 s without any stimulation. Afterwards, 10 stimulation pulses (wavelength 488 nm through the objective or 491 nm through the waveguide) with a frequency of 1 Hz and a duration of 60 ms are sent (red dashed line). Thus, the stimulation pulses are shifted relatively to the imaging time frames so that no overlap occurs (cf. close-up in Figure S5(b)). After the stimulation, the background is recorded for another 30 s.

Evaluation procedure of neural response

In order to detect neural response, fluorescence images with an exposure time of 10 ms and a sampling rate of 10 Hz are recorded during stimulation. Both Rhod-3 and mCherry (bound to ChR-2) lead to a baseline which is different for each pixel in the image. Thus, it is not reasonable to use absolute counts as a figure of merit for cell activity. In addition, the time signal of a single pixel is very noisy, as exemplary shown in Figure S7(b). This hampers the extraction of neural response especially

if it is weak. Thus, to extract weak spiking signals from the noisy data, only the time window from 21 s to 31 s in which the stimulation occurs is taken into account. On this data, we perform a fast Fourier transform (FFT). For an ideal signal with 10 consecutive exponentially decaying peaks of 1 Hz, the frequency domain exhibits distinct peaks for frequencies with integer multiples of 1 Hz, as plotted in Figure S6(a). As a figure of merit for a responding pixel, we add up amplitudes of 1 Hz, 2 Hz, 3 Hz, 4 Hz and (-)5 Hz and normalize the values to the pixel with the maximum value in the entire FOV. In this way, the DC baseline in the time domain is omitted.

This evaluation procedure works fine for a pronounced response as in the measurement with objective stimulation (Figure 4(a) in the main text). In order to further increase the contrast between responding and non-responding pixels for weak and noisy signals, the values of 3x3 pixels in the time domain is averaged, before the FFT is calculated (Figure S6(c)). This increases the signal to noise ratio in the final plot (Figure 4(b), Panel (iii)), but at the same time reduces resolution by a factor of 3.

Neuronal cell culture growing on photonic chip

Figure S8 shows a microscope image of a neuronal cell culture growing on a photonic chip featuring OrmoComp waveguides on the lower right side and without photonic structures on the upper left side. The cell density and neural growth is not affected by the photonic structures. Neurons are growing next to the waveguides, on top of the waveguides and even cross the photonic devices. This proves that the waveguide material OrmoComp is non-toxic for the neurons and that the topography does not disturb an unrestricted cell growth.

Figure S9 shows a single neuron overlapping with five different waveguides. Nonetheless, the interaction area with each waveguide constitutes only a minor part of the neuron, so that subcellular stimulation becomes possible. In addition, by addressing the individual waveguides subsequently or even simultaneously, multiple spatial and temporal stimulation patterns can be sent. If the waveguide dimensions are reduced to the nanometre scale, subcellular stimulation even at the level of a single synapse will become possible.

Simulation of evanescent field stimulation of neurons

In order to confirm that a stimulation of neurons which only partially overlap with the waveguide is possible, we carry out numerical simulations. In our previous work, we showed that an optical power in a silicon nitride waveguide of only a few microwatts is sufficient to successfully stimulate a neuron in contact with the waveguide surface via the evanescent field.[28] Here, we use the same model but adjust the waveguide parameters to those of the rib waveguides used in the experiments (optical material parameters of OrmoComp with n_OrmoComp = 1.527, waveguide dimensions with w = 4.5 µm, h = 300 nm and h_{slab} = 40 nm).

Figure S10 shows the resulting optical power threshold in the waveguide to evoke an action potential in the neuron for different waveguide offsets (a) and waveguide rotations (b). The chosen offset range from –50 µm to +50 µm is larger than the soma of the neuron model. Thus, only a few neurites interact with the evanescent field for large waveguide shifts. Moreover, only the soma overlaps with the waveguide for certain waveguide rotations. Nonetheless, the simulations show that the neuron fires an action potential when an optical power in the order of microwatts is guided in the waveguide (λ = 491 nm).

The results support the statement that our photonic platform enables a subcellular stimulation of neurons partially interacting with the evanescent field.

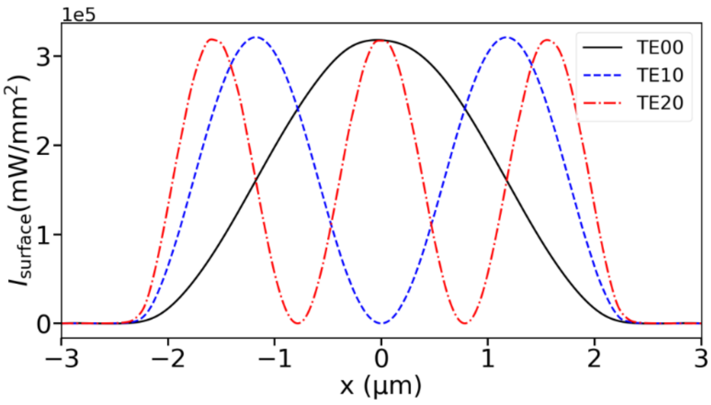

Figure S1: Computed power density at the surface level of the waveguide with the dimensions as shown in Figure 1(b) in the main text. The input power is set to 1 mW.

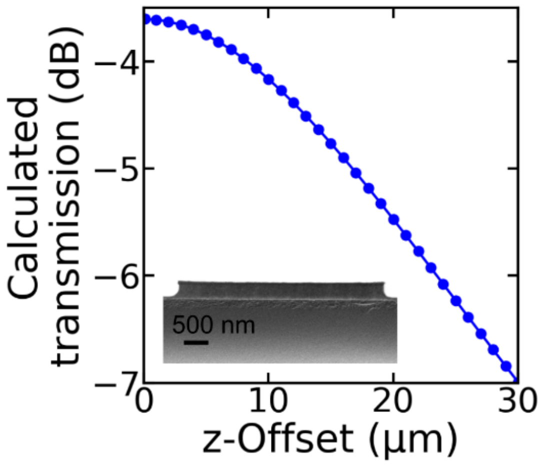

Figure S2: Calculated transmission derived from the overlap integral of a Gaussian beam and the fundamental TE-like mode of the used waveguide.

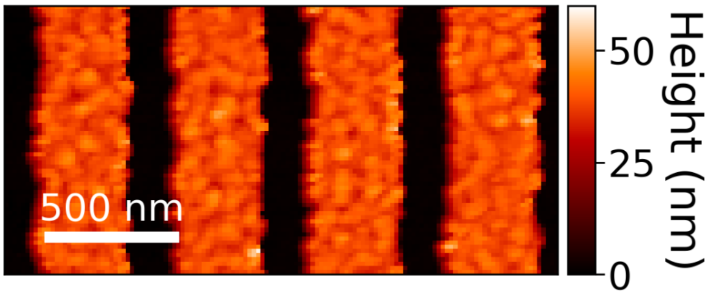

Figure S3: AFM measurement of 40 nm high Al grating on glass.

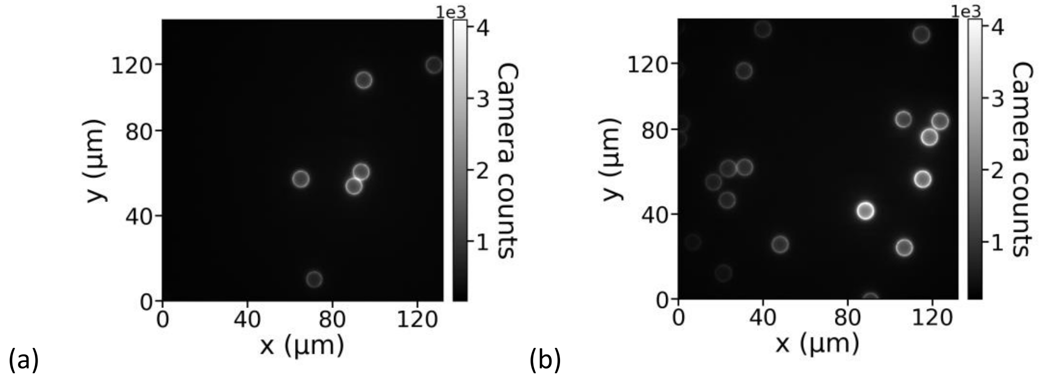

Figure S4: Fluorescence microscopy image of FITC-covered microbeads excited with 488 nm through the microscope objective in Epi-mode (cf. setup in Figure 3(a)). The focus is adjusted to the equatorial plane of the beads, so that they appear as a ring. The FOV shown in (a) corresponds to the objective based TIRF measurement in Figure 3(b), while the FOV shown in (b) corresponds to the evanescent field measurement in Figure 3(c).

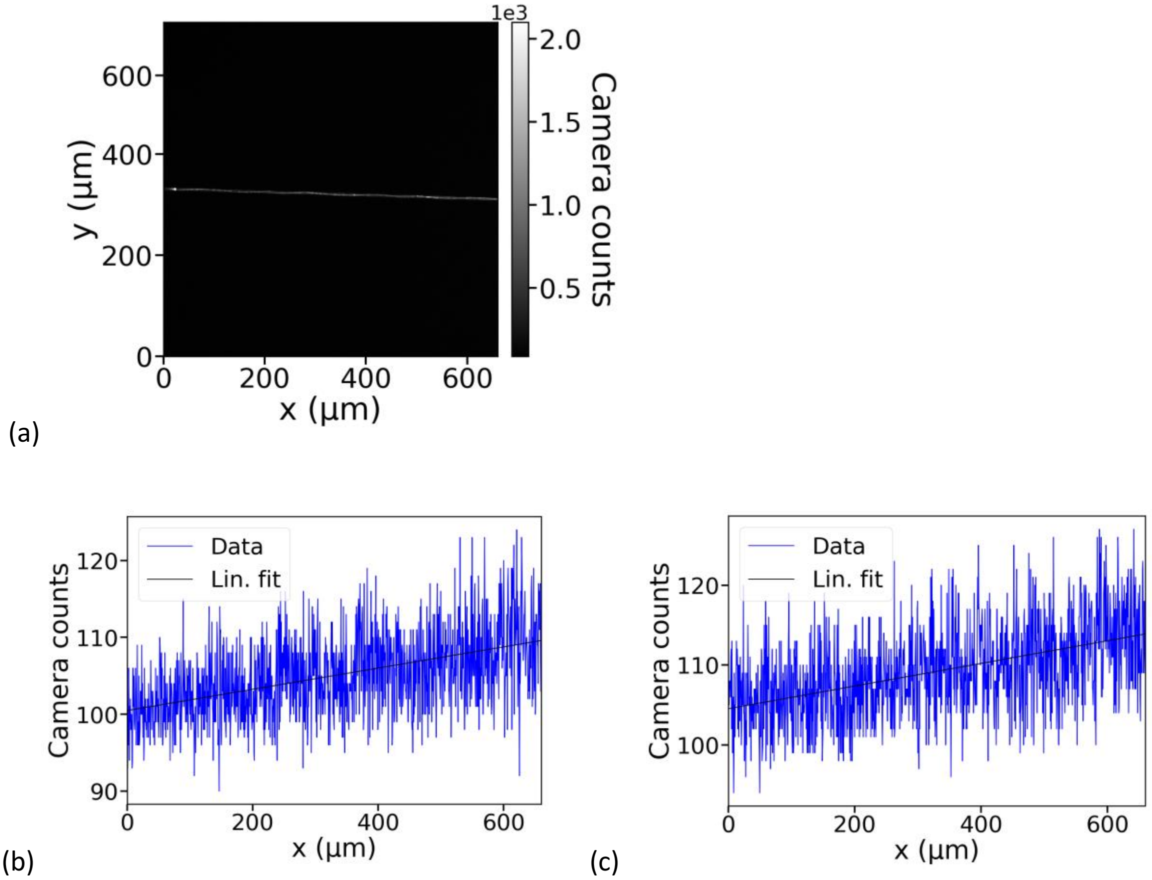

Figure S5: (a) Stimulation waveguide with light coupled in, while the excitation laser (561 nm) switched off so that no cells are visible. (b) and (c) show camera counts plotted along the *x* direction for *y* = 660 µm and 347 µm (a shade above the waveguide), respectively. Background noise is about 100 counts.

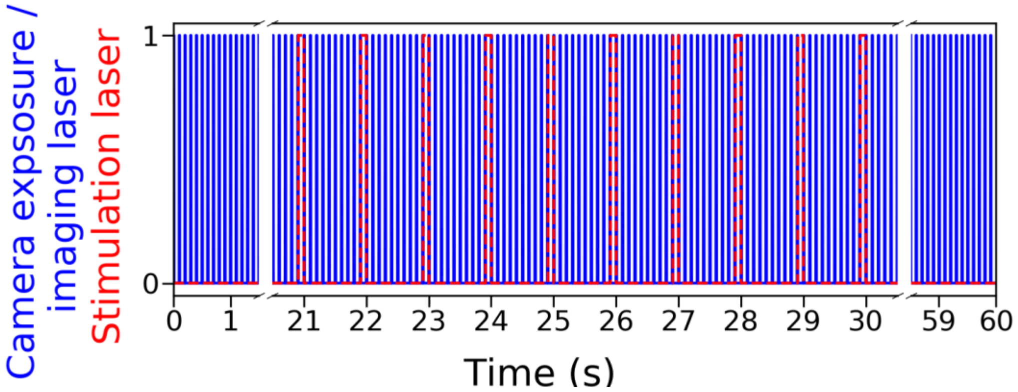

(a)

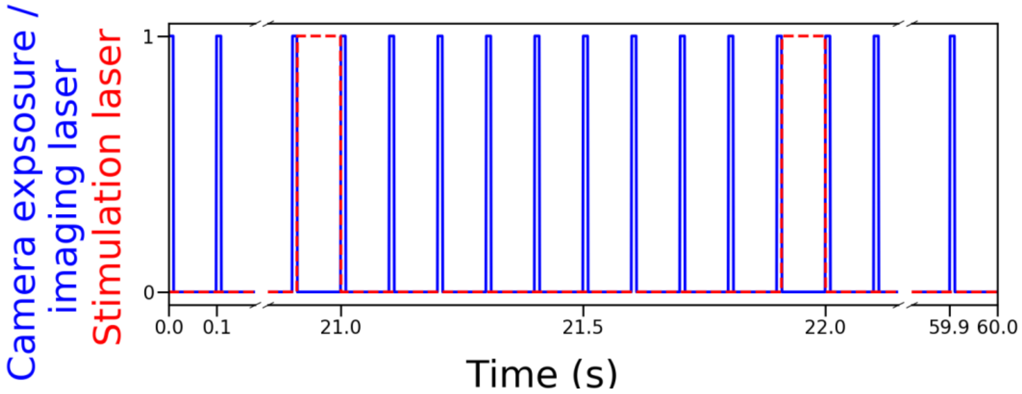

(b)

Figure S6: Measurement protocol of neural cell stimulation. (a) Overview and (b) close-up.

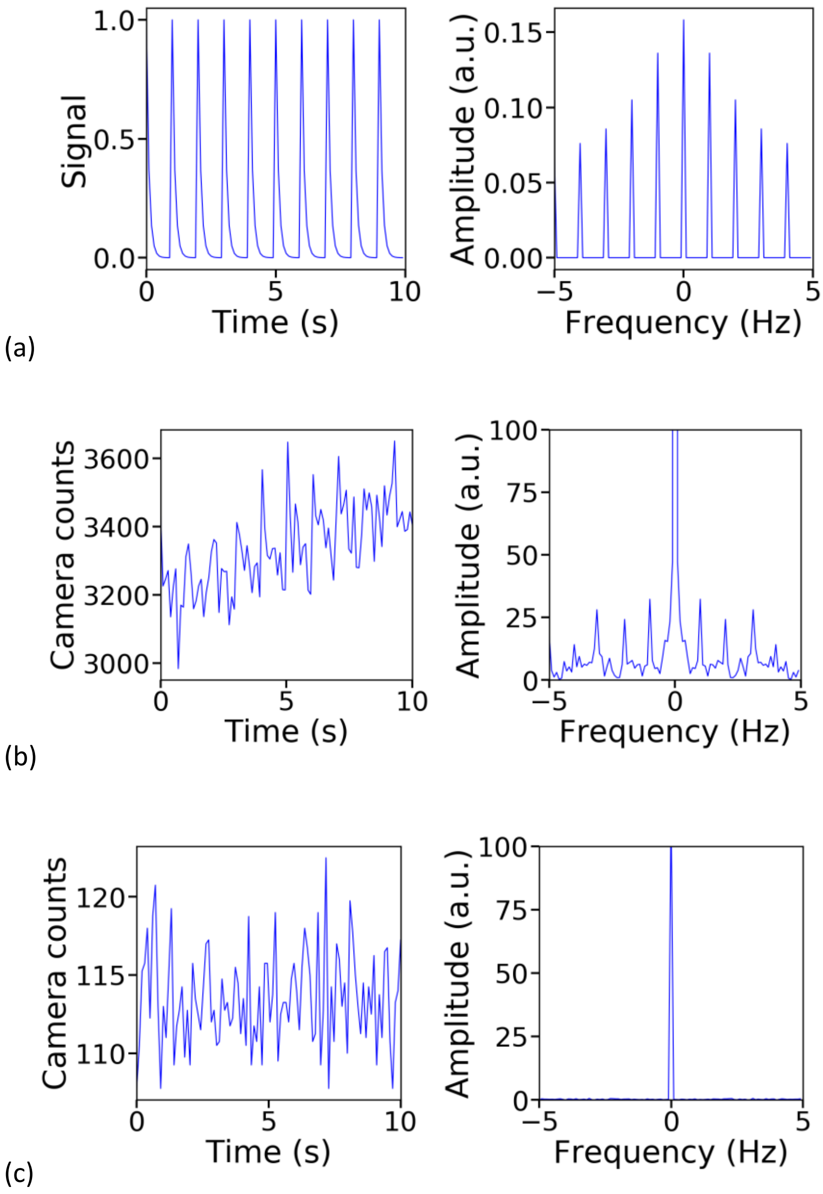

Figure S7: Time and frequency domain of (a) 10 consecutive exponential decaying functions with 1Hz, (b) an exemplary responding pixel of the measurement shown in Figure 4(b) in the main text and (c) an exemplary non-responding pixel.

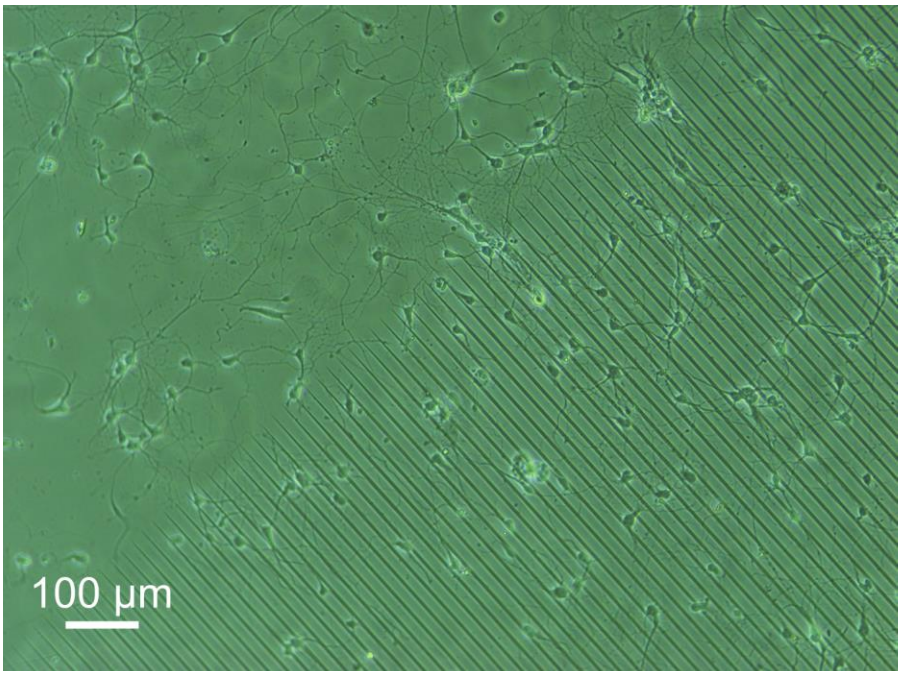

Figure S8: Neurons growing both next to and on top of the photonic waveguides.

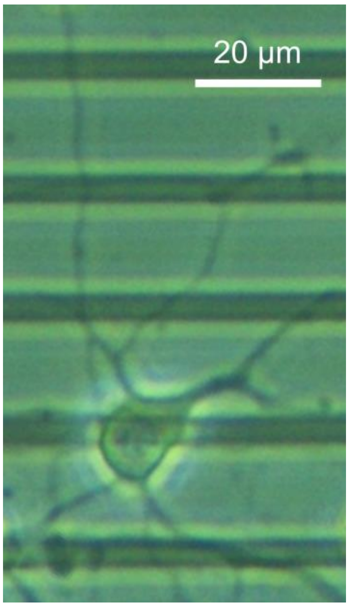

Figure S9: Single neuron overlapping with five different waveguides, which allows for flexible spatial and temporal stimulation patterns.

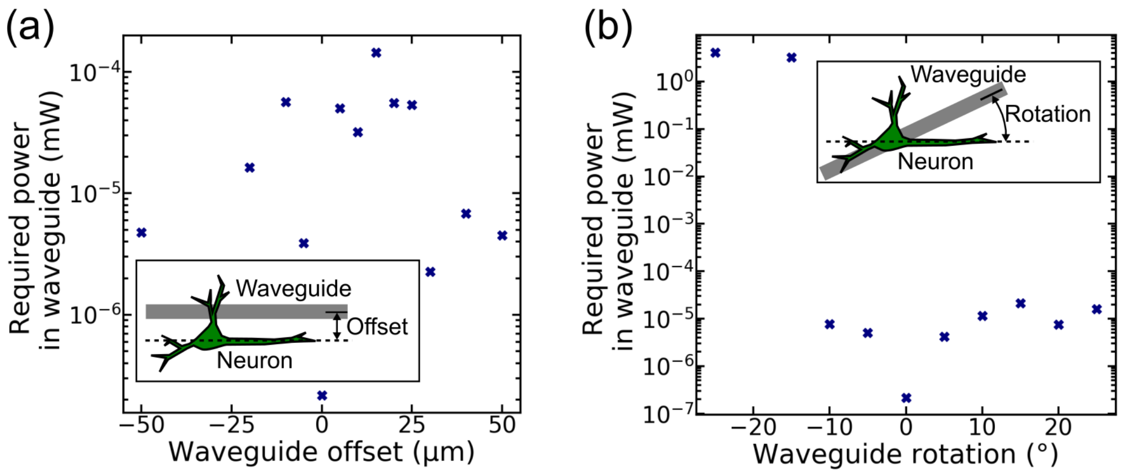

Figure S10: Optical power threshold in the waveguide to successfully stimulate a ChR-2 expressing neuron for different waveguide-neuron overlaps obtained by numerical simulation. (a) The waveguide is shifted relatively to the long axis of the neuron as shown in the inset. (b) The waveguide is rotated with the rotation centre located at the soma as shown in the inset. The simulation is based on a binary search algorithm to find the threshold power in the waveguide for successful neuron stimulation. The error threshold in the simulation is set to about 0.1 pW (error bars not visible in the plots). For details of the model, refer to our previous work. [28]
